# Comparative assessment of RNA-dependent RNA polymerase (RdRp) inhibitors under clinical trials to control SARS-CoV2 using rigorous computational workflow†

**DOI:** 10.1039/d1ra04460e

**Published:** 2021-09-02

**Authors:** Dweipayan Goswami

**Affiliations:** Department of Microbiology & Biotechnology, University School of Sciences, Gujarat University Ahmedabad 380009 Gujarat India dweipayan.goswami@gujaratuniversity.ac.in

## Abstract

The devastating effect of SARS-CoV2 continues and the scientific community is pursuing to find the strategy to combat the spread of the virus. The approach is adapted to target this virus with medicine in combination with existing vaccines. For this, the medications that can specifically inhibit an enzyme essential for viral replication ‘RNA-dependant-RNA polymerase (RdRp)’ of SARS-CoV2 are being developed. RdRp is the enzyme commonly found in all RNA viruses but is absent in humans. There are in total 60 different RdRp inhibitors already under clinical trials for combating other RNA viruses, which are sought to even work for SARS-CoV2. These inhibitors are classified as nucleoside/nucleotide analogues and nonnucleoside/nonnucleotide analogues. In this study, all the known RdRp inhibitors were computationally targeted in the native form and their active form making the use of molecular docking, MM-GBSA and molecular dynamics (MD) simulations to find the top two of each nucleoside/nucleotide analogues and nonnucleoside/nonnucleotide analogues. The results showed ribavirin 5′-triphosphate and favipiravir ribonucleoside triphosphate (favipiravir-RTP) to be the top two nucleotide analogues while pimodivir and dihydropyrazolopyridinone analogue 8d were the top two nonnucleosides/non-nucleotide analogues.

## Abstract 2

Identifying the inhibitors for RNA-dependant-RNA polymerase (RdRp) of SARS-CoV2.

## Graphical abstract

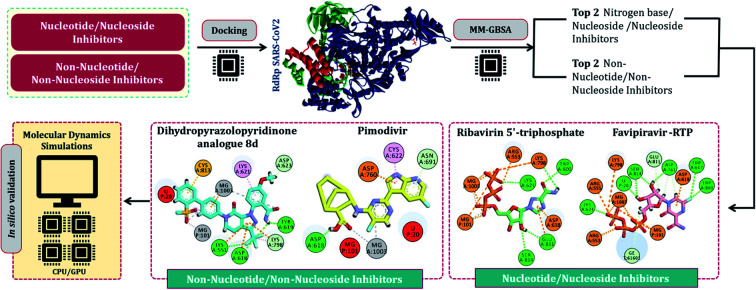

## Introduction

RNA viruses are notorious and have always challenged humans by causing infections that have a history to become disastrous.^1^ Most of the deadly RNA viruses either belong to the two main groups, positive (+) single-stranded (ss) RNA viruses or negative (−) ssRNA viruses. Norovirus, SARS-CoV, MERS-CoV, SARS-CoV2, hepatitis C virus, dengue virus, virus, poliovirus, influenza A, B and C viruses, Ebola virus, and many more viruses are classified as RNA viruses and have raised alarming situations for humans to combat.^2^ The recent of all, severe acute respiratory syndrome coronavirus-2 (SARS-CoV-2), which is thought to have evolved by the end of November 2019, has devastated humanity to such an extent that the pandemic has just raised in strength although vaccines being available for its combat. SARS-CoV2 belongs to a larger family of +ssRNA viruses, coronaviruses (CoV), named based on its morphology, which shows spikes on its outer surface making it resemble the sun-situated crown, the corona. These CoVs have four main phylogenetic branches based on their genetic sequences and they are, alpha (α), beta (β), gamma (γ) and delta (δ) CoVs. The viruses that can infect mammals fall in the α and β CoVs clads, while δ and γ CoVs thrive in aves.^3^ CoVs that can infect bats and humans are highly similar and are thought to have evolved from a common β CoV ancestor. There exist at least 100 more CoVs in bats that can become lethal by mutation or recombination gaining the ability to infect humans.^4^ Moreover, β CoVs have the ability to undergo frequent recombination led by raising activity at the human–animal interface. Cheng and colleagues back in 2007 said, lethal CoVs can evolve by stable genetic mutation and recombination, which is literally an ‘a ticking time bomb’ as a threat to humanity, which in turn is found to be true today.^5^

Vaccines developed to control SARS-CoV2 have acclaimed praises, however, there are several observed setbacks (i) vaccines not 100% efficient, (ii) the safety concerns are not well understood among various human races, and (iii) the efficacy of vaccines varies from person to person. Therefore, it is essential to have an alternative medication that can even work synergistically with existing vaccines.^6–8^ The one realistic option is oral and injectable inhibitors of RNA-dependant-RNA polymerase of SARS-CoV2 as a medication. This approach has the potential to work under stand-alone therapy and even along in combination with vaccines. RNA-dependant-RNA polymerase, abbreviated as ‘RdRp’ (from now onwards) is a unique enzyme found in RNA viruses and is absent in humans, and so it is considered an ideal target to design inhibitors for. Briefly, RNA viruses, possess RNA as genetic material and RdRp is the key enzyme that allows the virus to replicate and produce mRNAs of viral proteins in the host cell as follows. RNA viruses infect a host cell, their genetic material *i.e.*, viral RNA is immediately read as a template by host (human) ribosomes, which translate this viral RNA to produce RdRp, allowing the virus to hijack human cellular machinery. Then, RdRp immediately starts its function for synthesizing negative-strand subgenomic RNA, the synthesis of different structural protein-related mRNAs, and the replication of viral genomic RNA. Moreover, RdRp is highly efficient in its function, which allows RNA viruses to thrive in the host cell.^8–11^

For SARS-CoV2, a complex of the non-structural protein (nsp) 12, along with a heterodimer of nsp7-nsp8 accompanied by an additional nsp8, form the RdRp complex. This complex is designated as ‘nsp12-nsp7-nsp8’ and is considered the core RdRp polymerase.^10,12^ RdRp of different RNA viruses forms a diverse group of protein enzymes that perform identical functions in all these viruses, but at a genetic level, their sequence similarity tends to be as low as 30% from two distinct RNA viruses belonging to distant families. RdRp is a crucial viral protein that is perceived as an important therapeutic target since it plays a critical part in the replication of the RNA genome and in light of the fact that the human host does not have a structurally and functionally analogous comparable protein to this.^10,13^ Also, because of the lack of the existence of the protein identical to RdRp in mammalian cells, its inhibition does not cause side effects and does not interfere with normal mammalian metabolic pathways, and in this manner, RdRp is viewed as an appealing therapeutic target for drug development.^14^ Developing powerful RdRp inhibitors that have the potential to block viral replication for some time has been an examination theme for scientific avenues and researchers of academic and pharmaceutical care. There are two known classes of RdRp inhibitors: nucleoside simple inhibitors and nonnucleoside simple inhibitors. These two classes show contrasts in structure and have different modes of action. There are over 60 RdRp inhibitors developed so far for different RNA viruses, which are under clinical trials for their action on RdRp of SARS-CoV2 and are vividly described in the review published by Tian and colleagues.^11^ Most of the nucleoside inhibitors are sold as precursors (nitrogen bases and nucleosides) as they tend to get converted to nucleotide form by cellular enzymes, which is considered as the active form of inhibitor possessing the ability to bind to the active site of RdRp and in turn, blocking the viral replication process. While the non-nucleotide inhibitor does not require such activation and upon gaining entry into the cell, they directly bind at the active site or at the allosteric site of RdRp halting its normal functioning.^11,15^

**Fig. 1:**
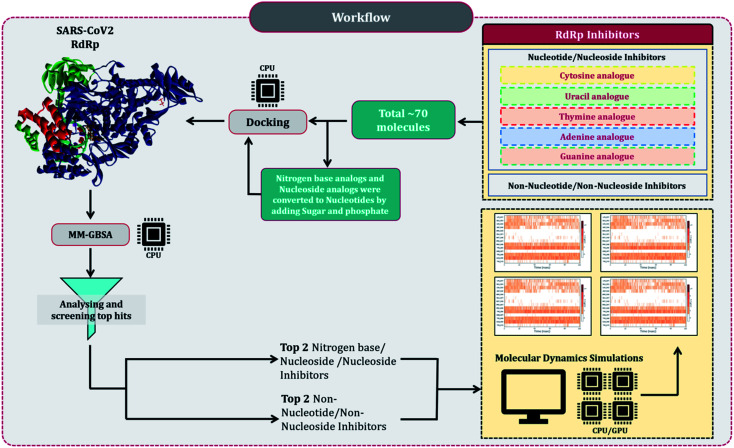
Schematic workflow explaining the rationale of the study.

Despite several RdRp inhibitors already being under clinical trials, their comparative assessment, at least a theoretical model is missing. The current work depicts the findings of *in silico* research to fill this loophole. Briefly, structures of all known molecules under clinical trials were retrieved using the drug CAS number from PubChem. The CAS numbers of these drugs were obtained from the review published by Tian and colleagues.^11^ For nitrogen bases analogues and nucleoside analogue inhibitors, structures of their active forms were retrieved from PubChem and a total library of 73 compounds was constructed, which included nitrogen bases analogues as inhibitors, nucleoside analogues as inhibitors and nonnucleoside/nonnucleoside inhibitors. The next step performed was to dock all these compounds with RdRp of SARS-CoV2 using molecular docking. For this step to occur accurately, the assessment of the active site of the target (RdRp) becomes very important. As the drugs used for the study were actually developed as RdRp inhibitors of other ssRNA viruses, assessment of their protein similarity to that of SARS-CoV2's becomes an important facet, therefore all the RdRp's of various distinct CoVs were superimposed over the reference 3D structure of RdRp of SARS-CoV2, allowing us to identify the active site, which was then used for docking. The results of docking were re-scored using the energy of the binding using MM-GBSA. The top two hits for each of nucleoside/nucleotide inhibitors and nonnucleoside/non-nucleotide were screened based on docking and MM-GBSA score assessment. All four hits were further validated for their stable interaction with RdRp of SARS-CoV2 using Molecular Dynamics (MD) simulations. The workflow of the *in silico* experimentations and assessments performed is represented in Fig. 1.

## Materials and methods

### Assessment RdRp from various ssRNA viruses for determination of active site

There are multiple different types of RdRp spread across various ssRNA viruses. Superimposing the representative structures of major +ssRNA viral families will provide a tentative idea regarding the active sites of the enzyme. This is essential to perform, as the RdRp inhibitors developed so far are for a particular viral RdRp type. If there is a structural similarity of SARS-CoV2 RdRp with other viral RdRp, then there is a high probability that an inhibitor developed for some other +ssRNA virus may also work for the SARS-CoV2 RdRp. A total of five different RdRp belonging to four different families of +ssRNA viruses were superimposed. For the superimposition, the main catalytic chain was used as it possesses the active site for the incoming nucleotide. Protein with PDB id, 7AAP, RdRp of SARS-CoV2 as a representative of Coronaviridae family, 2CKW-RdRp of norovirus as a representative of norovirus of Caliciviridae family, 4OOW-RdRp of hepatitis C virus as a representative of Flaviviridae family, 5K5M-RdRp of dengue as a representative of norovirus of Flaviviridae family and 4R0E-RdRp of poliovirus 1 as a representative of Picornaviridae family, were taken and were aligned with the matchmaker tool of UCSF Chimera v 1.14.^16^ This aligned all the proteins with the template reference of 7AAP, RdRp of SARS-CoV2. This was done to assure the catalytic site of all RdRps were identical. Moreover, the co-crystalized ligand favipiravir ribofuranosyl-5′-triphosphate (favipiravir-RTP) of 7AAP was falling in the common superimposed active site. Further, the entire study was performed with 7AAP protein^17^ and the docking coordinates were chosen to be the exact place where its co-crystallized ligand, favipiravir-RTP was situated.

### Ligand library, ligand preparation and protein preparation

Briefly, structures of all known molecules under clinical trials were retrieved using the drug CAS number from PubChem. For nitrogen bases and nucleoside inhibitors, their structure of active form was retrieved from PubChem and a total library of 72 compounds was constructed, which included nitrogen bases analogues as inhibitors, nucleoside analogues as inhibitors and non-nucleoside inhibitors. All the ligands were retrieved in the SDF format and were imported to Schrödinger Maestro for the ligand preparation for docking. Ligand preparation helps in generating the low energy structures and allows the option for expanding each input structure according to its desired stereochemistry by generating variations on ionisation state tautomer's ad ring confirmations. LigPrep wizard in Schrödinger Maestro was used to generate ionization states for each ligand structure with Epik^18,19^ at a physiological pH of 7.2 ± 0.2 unit. Rest other options were kept as default and the ligands were minimized under the OPLS2005 force field. The output files prepared on ligand minimization were used for docking using Glide.

Protein 7APP was retrieved from PDB, it is nsp7-nsp8-nsp12 SARS-CoV2 RNA-dependent RNA polymerase in complex with the template, primer dsRNA and favipiravir-RTP. It has a total of 4 chains, chain A is the main chain with the catalytic domain and is nsp12, chain B and D is nsp8, while chain C is nsp7.^17^ The protein was imported to Schrödinger Maestro and prior to docking, the protein was prepared in the protein preparation wizard of Maestro. Here, the protein was first pre-processed by adding hydrogens, converting selenomethionines to methionines and het states were generated in Epik for pH 7.0. In the next step of the protein preparation, the H-bond assignment was performed using PROPKA for pH 7.0 for optimizing the protein. Once the protein was optimized, the restrained minimization of protein was performed using OPLS-2005 (Optimized Kanhesia for Liquid Simulations) force field.^20–22^ All the chains were kept, and the RNA molecule was also kept. These tasks were all performed using the Protein Preparation Wizard of Schrödinger Maestro.^23,24^

### Molecular docking

The optimized and minimized protein from the previous step was used for docking. The first step for docking is to prepare the grid at the exact same coordinates as that of the native ligand (favipiravir-RTP) in 7APP. The grid box of the size 13 Å × 13 Å × 13 Å was prepared at the coordinates with the center position defined as the following coordinates, 96.9° on *X*-axis, 97.1° on *Y*-axis and 111.7° on *Z*-axis. The grid for docking with the mentioned parameters was prepared using the ‘receptor grid generation’ feature of the Glide module in Schrödinger Maestro. For docking, the output file of (i) receptor grid generation and (2) prepared minimized ligands were imported in the ‘Ligand docking’ window of Glide module in Schrödinger Maestro. Under the settings, the precision of docking was set as ‘Extra Precision (XP)’, ligand sampling was set as ‘Flexible’ and the Epik state penalties were added to the docking score. The output was set to show only the best pose. The entire docking was performed using the Glide module in Schrödinger Maestro.^23,24^ The entire output file of docking was used to perform an MM-GBSA assessment.

### MM-GBSA calculation

The endpoint Δ*G* was calculated using Molecular Mechanics Generalized Born Surface Area (MM-GBSA), which is a semi-quantitative technique to evaluate ligand–receptor binding.^25–28^ The output file of all the docked complexes so obtained after performing XP docking was used for MM-GBSA calculations. The file was imported to the Prime wizard of Schrödinger Maestro's where it was optimized.^29^ The binding energy change for a group of receptors and ligands was calculated using the OPLS-2005 force field. The Δ*G* binding energy transition was calculated using the following equation:

1 Δ*G*Bind = Δ*E*MM + Δ*G*Solv + Δ*G*SA

Here, Δ*G*Bind stands for the binding of receptor and ligand molecules in the solution as the molar Gibbs energy. Δ*E*MM is the variance between the minimized energy of the protein–ligand complexes, while Δ*G*Solv is the sum of the solvation energies for the protein and ligand and the variation between the GBSA solvation energy of the same. Δ*G*SA is the difference in the surface area energies for the complexes. After assessing the docking score and Δ*G*Bind score of MM-GBSA, the top two hits for each of nucleoside/nucleotide inhibitors and non-nucleoside/non-nucleotide were screened. The total of the top four RdRp inhibitors so screened were further validated using MD simulations.

### Molecular dynamic (MD) simulation

The simulations for the top two of each, nucleoside/nucleotide inhibitors and nonnucleoside/nonnucleotide were performed for a period of 100 ns each for the docked complex using Desmond package (Schrödinger Release 2018-4).^30^ Initially, the energy minimization of the protein–ligand complex was performed using the OPLS-2005 force field, after which the system was built using the TIP3P solvent model, which specifies a 3-site rigid water molecule with charges and Lennard-Jones parameters assigned to each of the 3 atoms. Periodic boundary conditions (PBC) were set up by selecting the orthorhombic shape simulation box fitting the protein–ligand complex having 15 Å buffer space around the periphery of the protein. Followed by neutralisation with the placement of Na^+^ ions and salt concentration of 0.15 M Na^+^ and Cl^−^ counter ions to simulate the background salt and physiological conditions using OPLS-2005 force fields. Once the system gets incorporated, MD simulation was performed using NPT (constant number of particles, pressure, and temperature) ensemble with 300 K temperature and 1.013 bar atomic pressure and default surface tension using a smooth particle mesh ewald (PME) method to calculate long-range electrostatic interaction potential energies. The MD simulation was executed for a period of 100 ns for each of the complexes and 1000 number of frames of trajectories were recorded. On completion of the simulation, each trajectory was analysed in simulation interaction diagram wizard, which computes trajectories for Root Mean Square Deviation (RMSD) and Root Means Square Fluctuation (RMSF). Protein–ligand contact profiles for crucial interacting amino acid residues and the timeline of these specific interactions are also computed with respect to 100 ns simulation. The validation of docking poses and interactions predicted by inhibitors with amino acids during docking was performed using the procedure of MD simulations.

## Results and discussion

### Assessment RdRp from various ssRNA viruses for determination of active site

**Fig. 2:**
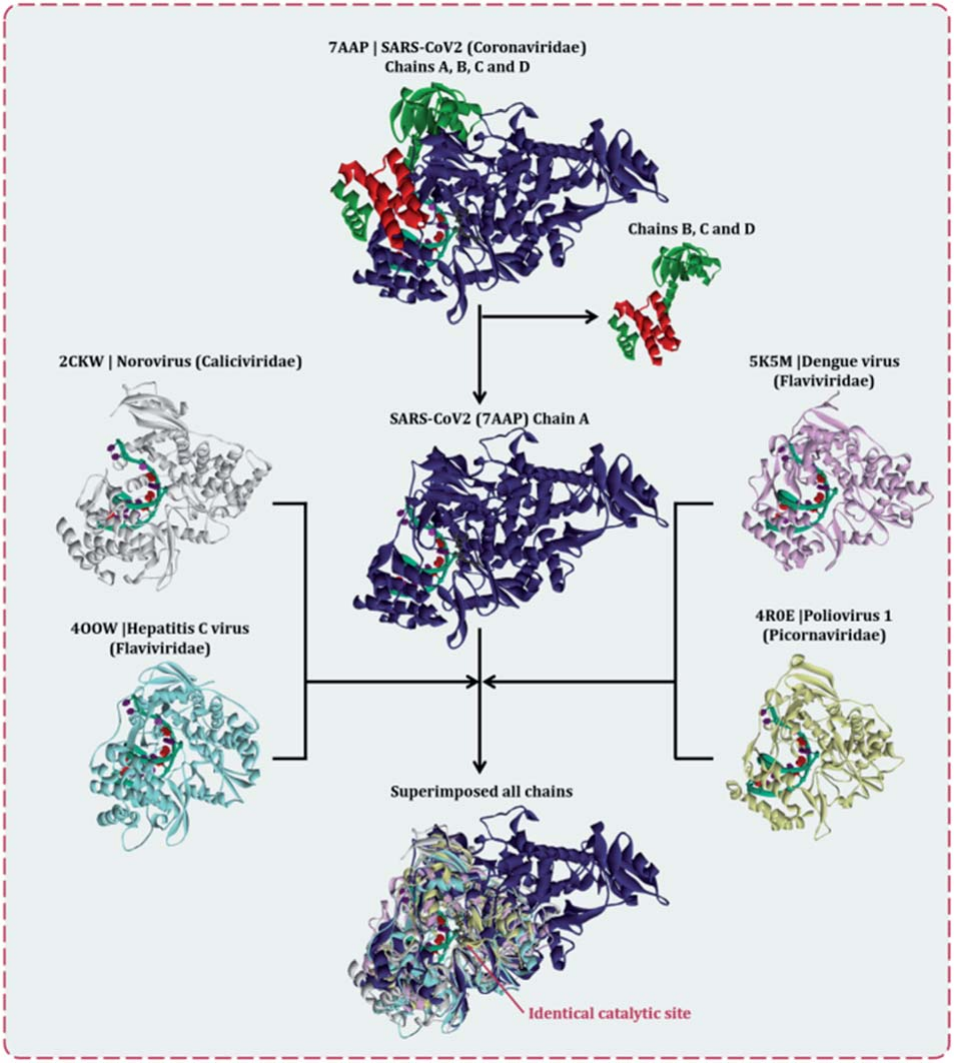
Superimposition of RdRps from various +ssRNA viral family representative viruses to understand the structural similarity amongst various of same enzyme from different viral families, the penultimate superimposed image shows identical active site.

**Fig. 3:**
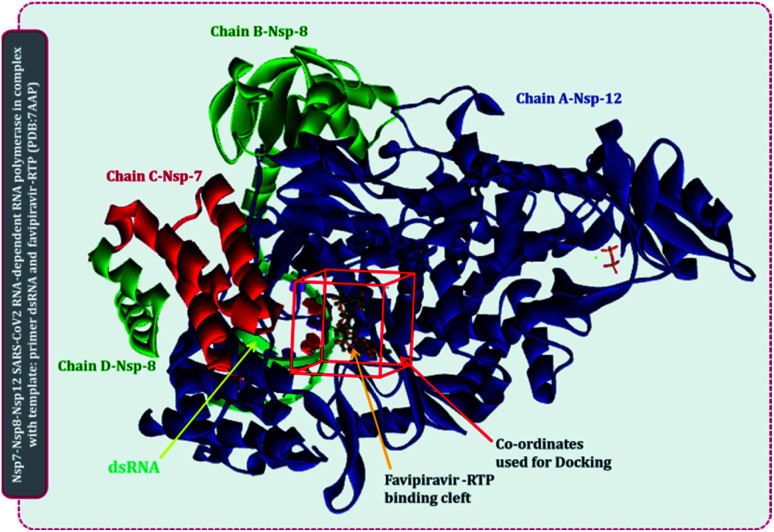
Representation of SARS-CoV2 RdRp complex with primer RNA (PDB id 7AAP) with site of co-crystallized ligand flavipiravir-RTP at its catalytic site and site used for docking for current study.

Several lethal viruses such as norovirus, SARS-CoV, MERS-CoV, SARS-CoV-2, hepatitis C, dengue virus, zika virus, poliovirus, and venezuelan equine encephalitis virus are +ssRNA viruses. Efforts are being made to develop inhibitors of RdRp of all these viruses. RdRp shares an adequate amount of structural similarity, especially in the region of the active site, which is also the catalytic site.^11,31^ Phylogenetic analysis and sequence similarity search represented RdRp of Coronaviridae are much more genetically related to Flaviviridae (Fig. S1, ESI†). Similar findings with a much deeper understanding regarding the genetic evaluation of RdRps is represented by Venkataraman and colleagues,^32^ which ultimately explains that there is a lot of structural and functional similarities among the RdRp of various viral families. Moreover, a different group of researchers is trying to develop an inhibitor for each of these RdRps of different viruses. For instance, cytidine triphosphate (CTP) is reported to inhibit the RdRp of influenza viruses, fluorinated nucleosides inhibitors are known to inhibit RdRp of hepatitis virus C, sofosbuvir is known to inhibit RdRp of Zika virus and chikungunya virus, favipiravir is reported to inhibit RdRp of influenza A&B viruses and Ebola virus.^11^ In the event where there is an underlying likeness in the RdRp structure of SARS-CoV2 with other viral RdRp, at that point there is a high likelihood that an inhibitor created for other RNA virus may likewise work for the SARS-CoV2. A sum of five distinctive RdRp having a place with four unique groups of +ssRNA viruses were superimposed. For the superimposition, the primary chain was utilized as it has the catalytic site for the approaching nucleotide. Protein with PDB id, 7AAP, RdRp of SARS-CoV2 as a representative of Coronaviridae family, 2CKW-RdRp of norovirus as a delegate of Caliciviridae family, 4OOW-RdRp of hepatitis C infection as a delegate of Flaviviridae family, 5K5M-RdRp of dengue as an agent of norovirus of Flaviviridae family and 4R0E-RdRp of poliovirus 1 as an agent of Picornaviridae family, were taken and superimposed (Fig. 2). The superimposition showed that despite possessing structural differences, catalytic active sites of all the proteins were identical. Moreover, Multiple Sequence Alignment (MSA) of all these proteins were also performed, which showed that there exists around ∼30% sequence similarity amongst each other, but although that, their structures superimposed, showing the cavity of the active site being highly identical. Moreover, the RdRp, of SARS-CoV2 had co-crystallized ligand as, favipiravir-RTP (PDB id 7AAP), located in the identical superimposed active site. Further, the entire study was conducted using 7AAP protein^17^ and the docking coordinates were chosen to be the exact place, where its co-crystalized ligand, favipiravir-RTP was situated (Fig. 3). Moreover, the reason for using 7AAP for the rest of the study was that it represents the most accurate structure with co-crystallized inhibitor. 7AAP consists of the entire complex of nsp7-nsp8-nsp9 with primer RNA duplex along with favipiravir-RTP. The structure of this protein was determined using electron cryomicroscopy to a resolution of 2.5 Å.^17^ There are reports, where the same protein with different PDB id has been used for bioinformatic studies, which are 6NUR and 7BW4.^15,33,34^ However, these RdRp structures do not possess any co-crystallized inhibitor, had lower resolution than 7AAP, and lacked a chain or two, therefore the protein 7AAP was chosen to perform all the following studies.

### Molecular docking and MM-GBSA calculations

**Fig. 4:**
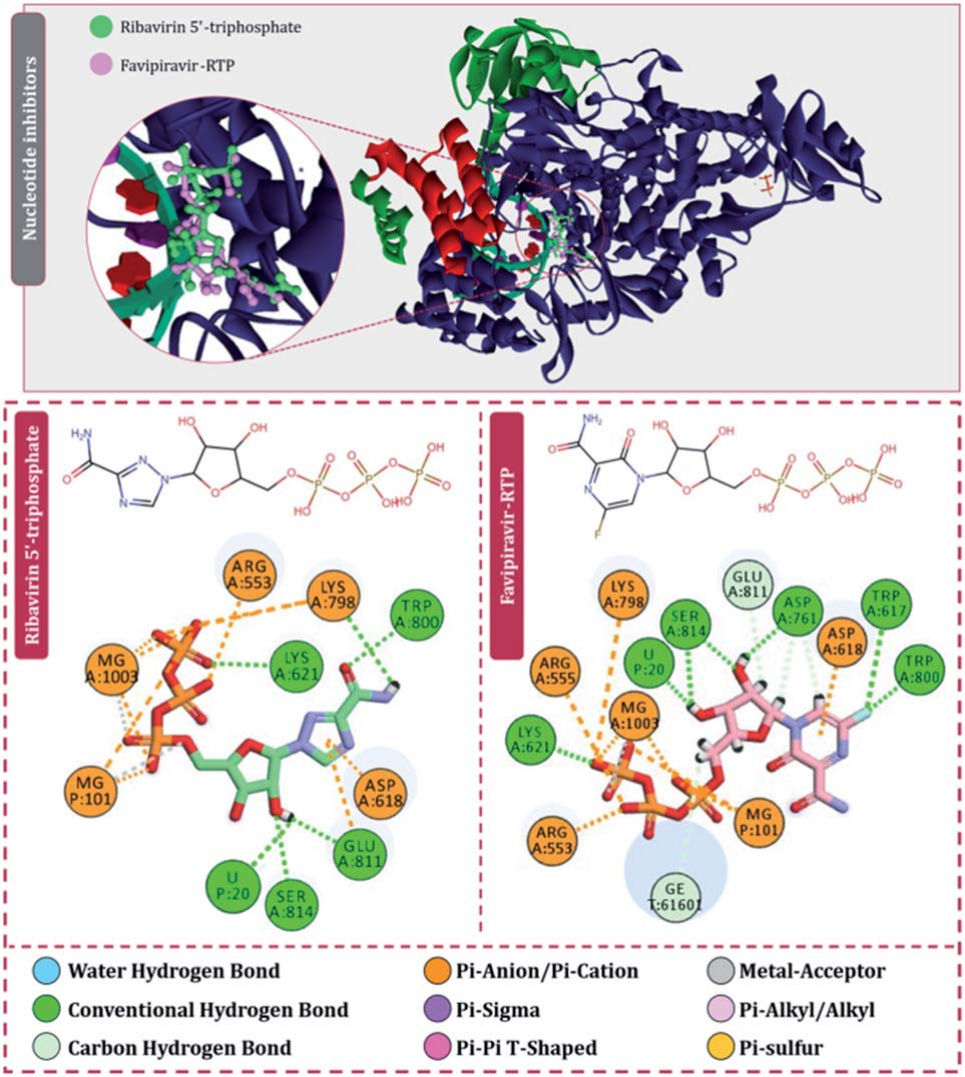
Interaction of nucleoside/nucleotide analogues, ribavirin 5′-triphosphate and favipiravir-RTP with RdRp of SARS-CoV2.

For the interaction of RdRp of SARS-CoV2 with the drugs in clinical trials, the docking locus as represented in Fig. 3 was chosen, the structures of all RdRp inhibitors under clinical trials were retrieved using the drug CAS number from PubChem with reference to the review published by Tian and colleagues.^11^ For nitrogen bases and nucleoside inhibitors, their active form structures were retrieved from PubChem and a total library of 73 compounds was constructed, which included nitrogen bases analogues as inhibitors, nucleoside analogues as inhibitors and nonnucleoside inhibitors. The docking scores of all these compounds in their best pose with minimum RMSD values are represented in Table 1 and the compounds are arranged in the order of ranks from best to worst. As per the rationale of the study, the top two hits for each of nucleoside/nucleotide inhibitors and nonnucleoside/nonnucleotide were chosen for further assessment. From the docking assessment, the top two nucleoside/nucleotide inhibitors were found to be ribavirin 5′-triphosphate and favipiravir-RTP, and their interaction with RdRp is represented diagrammatically in Fig. 4. It is observed that the orientations at with both these compounds interact with RdRp is identical, where the tail phosphate oxygen interacts with Lys621. Nitrogen-containing rings of both these drugs interact identically with Asp618 by making pi–anion interactions. Trp800 and Ser814 form hydrogen bonds with both the analogues nucleotide analogues. The binding energy of ribavirin 5′-triphosphate is found to be slightly better than that shown by favipiravir-RTP, where these values being −7.41 kcal mol^−1^ and −7.36 kcal mol^−1^, respectively. Nucleoside/nucleotide inhibitors are further classified as pyrimidine nucleoside/nucleotide analogues, purine nucleoside/nucleotide analogues and lastly miscellaneous nucleoside/nucleotide analogues and surprisingly, both our top hits, ribavirin 5′-triphosphate and favipiravir-RTP belong to the class of miscellaneous nucleoside/nucleotide analogues.^11^ It is accounted that ribavirin can straightforwardly incite antiviral action against various RNA viruses by the increasing recurrence of mutations in RNA viral genomes.^35^ The treatment of hepatitis C and viral haemorrhagic fevers by this compound has principally shown efficient outcomes. There are reports where viral mRNA guanylyltransferase and mRNA 20-*O*-methyltransferase of dengue virus being successfully inhibited by ribavirin 5′-triphosphate^36^ and was utilized as a remedial medication in the SARS flare-up in 2003.^37^ Tong and colleagues analysed ribavirin in combination with other medicinal therapies for patients with extreme COVID-19 symptoms and found that ribavirin treatment is not related to an improved negative change time in the SARS-CoV-2 test or with an improved death rate in patients with serious COVID-19, these outcomes however cannot directly suggest the ineffectiveness of this drug as the patients tested had extreme symptoms and therefore may be compromised severely with secondary infections.^38^ Also, a preliminary clinical examination considered the adequacy and effectiveness of the combined treatment consisting of the mix of interferon β-1b, lopinavir–ritonavir, and ribavirin for the treatment of patients with SARS-CoV2 infection. Studies in clinical trials revealed that early triple antiviral treatment was much more effective than a dual mix of lopinavir–ritonavir in reducing manifestations and shortening the span of viral shedding and clinic stay in patients with less severe symptoms and had minimal side effects which were easily managed.^39^

**Table 1:** List of compounds used under study, their classification, and their binding energy

Rank	Compound	Molecule ID	Classification	Binding energy (kcal mol^−1^)
1^a^	Ribavirin 5′-triphosphate	PubChem CID: 122108	Miscellaneous nucleoside	**−**7.41
2^a^	Favipiravir triphosphate (RTP)	PubChem CID: 5271809	Miscellaneous nucleoside	−7.36
3	2′-*c*-Methylcytidine triphosphate	PubChem CID: 15940324	Pyrimidine nucleoside	−7.19
4^b^	Pimodivir	CAS #: 1629869-44-8	Non-nucleoside inhibitors	**−**7.18
5	Galidesivir triphosphate	PubChem CID: 146047139	Purine nucleoside	−7.12
6^b^	Dihydropyrazolopyridinone analogue 8d	PubChem CID: 23646185	Non-nucleoside inhibitors	**−**6.98
7	Dihydropyrazolopyridinone analogue 8b	PubChem CID: 23646183	Non-nucleoside inhibitors	−6.93
8	Remdesivir triphosphate	PubChem CID: 56832906	Purine nucleoside	−6.88
9	Favipiravir	CAS #: 259793-96-9	Miscellaneous nucleoside	−6.87
10	Dihydropyrazolopyridinone analogue 8a	PubChem CID: 23646182	Non-nucleoside inhibitors	−6.76
11	N4-Hydroxycytidine 5′-triphosphate	PubChem CID: 147591	Pyrimidine nucleoside	−6.54
12	Grazoprevir	PubChem CID: 44603531	Non-nucleoside inhibitors	−6.34
13	Radalbuvir	CAS #: 1314795-11-3	Non-nucleoside inhibitors	−6.28
14	Benzimidazole analogue, 7g	PubChem CID: 44143448	Non-nucleoside inhibitors	−6.24
15	Setrobuvir	CAS #: 1071517-39-9	Non-nucleoside inhibitors	−6.23
16	Deleobuvir	CAS #: 1221574-24-8	Non-nucleoside inhibitors	−6.22
17	PSI-6130	CAS #: 817204-33-4	Pyrimidine nucleoside	−6.21
18	AL-335	CAS #: 1613589-09-5	Pyrimidine nucleoside	−6.21
19	Ribavirin	CAS #: 36791-04-5	Miscellaneous nucleoside	−6.21
20	ALS-8112	CAS #: 798009-58-2	Pyrimidine nucleoside	−6.11
21	PSI-7851	CAS #: 1064684-44-1	Pyrimidine nucleoside	−6.1
22	VCH-759	CAS #: 713139-25-4	Nonnucleoside inhibitors	−5.92
23	Nuc	CAS #: 1191237-69-0	Purine nucleoside	−5.9
24	PSI-7976	CAS #: 1190308-01-0	Pyrimidine nucleoside	−5.82
25	VX-135	CAS #: 798007-79-1	Pyrimidine nucleoside	−5.78
26	2′-*c*-Methylcytidine	CAS #: 20724-73-6	Pyrimidine nucleoside	−5.76
27	Sofosbuvir	CAS #: 1190307-88-0	Pyrimidine nucleoside	−5.76
28	Lumicitabine	CAS #: 1445385-02-3	Pyrimidine nucleoside	−5.73
29	Benzimidazole analogue, 7e	PubChem CID: 44143432	Non-nucleoside inhibitors	−5.68
30	JNJ-54257099	CAS #: 1255860-33-3	Pyrimidine nucleoside	−5.67
31	Dasabuvir	CAS #: 1132935-63-7	Non-nucleoside inhibitors	−5.67
32	MK-3281	CAS #: 886043-45-4	Non-nucleoside inhibitors	−5.66
33	Filibuvir	CAS #: 877130-28-4	Non-nucleoside inhibitors	−5.66
34	JTK-109	CAS #: 480462-62-2	Non-nucleoside inhibitors	−5.65
35	PSI-7672	CAS #: 1015255-46-5	Pyrimidine nucleoside	−5.63
36	Biphenyl diamine analogue, 20	PubChem CID: 25218554	Non-nucleoside inhibitors	−5.62
37	Aminophenol analogue, 6	PubChem CID: 25158538	Non-nucleoside inhibitors	−5.59
38	EIDD-2801	CAS #: 2349386-89-4	Pyrimidine nucleoside	−5.52
39	PSI-6206	CAS #: 1064684-44-1	Pyrimidine nucleoside	−5.45
40	NHC	CAS #: 3258-02-4	Pyrimidine nucleoside	−5.44
41	Mericitabine	CAS #: 940908-79-2	Pyrimidine nucleoside	−5.43
42	INX-189	CAS #: 1234490-83-5	Purine nucleoside	−5.43
43	Valopicitabine	CAS #: 640281-90-9	Pyrimidine nucleoside	−5.42
44	ACH-3422	CAS #: 798779-31-4	Pyrimidine nucleoside	−5.41
45	VCH-916	CAS #: 1200133-34-1	Non-nucleoside inhibitors	−5.28
46	IDX-375	CAS #: 1256735-81-5	Non-nucleoside inhibitors	−5.28
47	Benzimidazole analogue, 5a	PubChem CID: 44143438	Non-nucleoside inhibitors	−5.28
48	Benzimidazole analogue, 7m	PubChem CID: 44143453	Non-nucleoside inhibitors	−5.2
49	Aminothiazole analogue, 32	PubChem CID: 16068523	Non-nucleoside inhibitors	−5.2
50	Benzimidazole analogue, 7l	PubChem CID: 44143452	Non-nucleoside inhibitors	−5.17
51	BI 2536 analogue, 1b	PubChem CID: 11511524	Non-nucleoside inhibitors	−5.17
52	IDX-184	CAS #: 1036915-08-8	Purine nucleoside	−5.16
53	HCV-371	CAS #: 675184-27-7	Non-nucleoside inhibitors	−5.14
54	Tegobuvir	CAS #: 1000787-75-6	Non-nucleoside inhibitors	−5.14
55	Benzimidazole analogue, 5c	PubChem CID: 44143440	Non-nucleoside inhibitors	−5.14
56	Lomibuvir	CAS #: 1026785-55-6	Non-nucleoside inhibitors	−5.11
57	Nesbuvir	CAS #: 1132935-63-7	Non-nucleoside inhibitors	−5.11
58	Benzimidazole analogue, 5b	PubChem CID: 44143439	Non-nucleoside inhibitors	−5.11
59	4′-Azido-2′-deoxy-2′-*C*-methylcytidine	CAS #: 1019639-20-3	Pyrimidine nucleoside	−4.88
60	TMC-649128	CAS #: 1019639-33-8	Pyrimidine nucleoside	−4.76
61	Galidesivir	CAS #: 249503-25-1	Purine nucleoside	−4.69
62	Remdesivir	CAS #: 1809249-37-3	Purine nucleoside	−4.64
63	Benzimidazole analogue, 7h	PubChem CID: 44143433	Non-nucleoside inhibitors	−4.35
64	GSK-625433	CAS #: 885264-71-1	Non-nucleoside inhibitors	−4.34
65	Benzimidazole analogue, 7n	PubChem CID: 44143454	Non-nucleoside inhibitors	−4.34
66	Beclabuvir	CAS #: 958002-33-0	Non-nucleoside inhibitors	−4.33
67	ABT-072	CAS #: 1132936-00-5	Non-nucleoside inhibitors	−4.33
68	AT-527	CAS #: 2241337-84-6	Purine nucleoside	−4.32
69	BILB-1941	CAS #: 494856-61-0	Non-nucleoside inhibitors	−4.32
70	Benzimidazole analogue, 7a	PubChem CID: 44143444	Non-nucleoside inhibitors	−4.28
71	Kinome_3461	PubChem CID: 25263111	Non-nucleoside inhibitors	−4.28
72	Aminothiazole analogue, 21	PubChem CID: 16068527	Non-nucleoside inhibitors	−4.28

a The top two chosen nucleoside/nucleotide analogues as inhibitors for further studies.

b The top two chosen nonnucleoside/non-nucleotide analogues as inhibitors for further studies.

The second most effective inhibitor predicted based on docking scores under this study is favipiravir (Table 1). This drug is also a miscellaneous nucleotide precursor, which has proven to inhibit RdRp's of various RNA viruses and is thought to do wonders for SARS-CoV2. Favipiravir was originally developed for inhibiting RdRp of influenza A and influenza B and has shown tremendous success in infections caused in patients with these viruses and was approved in 2014 for its oral use. Favipiravir serves as a precursor of nucleotide analog, which on metabolizing in the human body is converted to its active form, favipiravir-RTP. There are reports suggesting the effective inhibition of RdRp of Ebola virus and rabies virus under clinical trials. Wang and colleagues^40^ showed that favipiravir can combat infection caused by SARS-CoV2 under *in vitro* investigations by its mode of action being inhibition of RdRp. A detailed study by Naydenova and colleagues^17^ showed that favipiravir-RTP binds to the RdRp of SARS-CoV2, but the interaction seems to be inferior and slower than that observed with RdRp of influenza viruses, but despite its slow interaction with RdRp of SARS-CoV2, the drug still has potentials to induce several mutations in the RNA genome of SARS-CoV2. The clinical trials have shown that the patients infected with SARS-CoV2 have an accelerated recovery when administered favipiravir compared to the un-treated control group, where the patients treated with favipiravir showed a recovery rate being shortened up to 30%. The favipiravir-treated group of patients further showed shortened virus clearance time, with improved chest CT scans. Furthermore, favipiravir is also proven to be effective for patients having underlying hypertension and/or diabetes.^14^ Stage three clinical trials of favipiravir for the treatment of hospitalized patients with SARS-CoV2 are on their way at the global stage. Future clinical trials are still inevitable to help us check the viability and wellbeing of favipiravir as a medication for the treatment of COVID-19.^14^

Based on our study, 2′-*c*-methylcytidine triphosphate, galidesivir triphosphate and remdesivir triphosphate are the third, fourth and fifth-best nucleoside/nucleotide analog inhibitors (Table 1). 2′-*c*-Methylcytidine is known to inhibit NS5B polymerase of hepatitis C virus, this drug upon phosphorylation into 2′-*c*-methylcytidine triphosphate, inhibits viral RNA chain elongation and viral RdRp activity of hepatitis C virus.^41^ Moreover, this compound has impressive pharmacokinetic and toxicokinetic profiles.^42^ Adenosine nucleoside analogue, galidesivir was originally developed to inhibit RdRp of hepatitis C virus by BioCryst Pharmaceuticals.^43^ Later this drug was also found to be effective against the Ebola virus. Now, based on clinical trials, this drug is also found to be effective in inhibiting RdRp of SARS-CoV, MERS-CoV, and SARS-CoV2. The developer of this drug, BioCryst Pharmaceuticals is currently undertaking several clinical trials for assessing its effectiveness against SARS-CoV2. In a study by Elfiky,^44^ galidesivir showed the docking energy with RdRp of SARS-CoV2 to be −7.0 kcal mol^−1^, while in the current study, this drug showed the docking energy to be −7.12 kcal mol^−1^ (Table 1), close to what was described in the literature. Remdesivir is known to be effective in inhibiting the RdRp in its native as well as phosphorylated form. However, in our study, the non-phosphorylated form shows a poor docking score with RdRp of SARS-CoV2 (Table 1), while the triphosphate form is only ranked as the fifth top nucleotide/nucleoside analog inhibitor. This drug is investigated in detail for its effectiveness in the suppression of RdRp of SARS-CoV2 in past one year, though this drug was originally found to be very effective against the Ebola virus.^9,10,45,46^

Despite several claims and positive results of remdesivir for treating SARS-CoV2, in current study it is only ranked fifth of the top nucleoside/nucleotide analogs, while it ranked eight among all the drugs used for the computational assessment (Table 1).

**Fig. 5:**
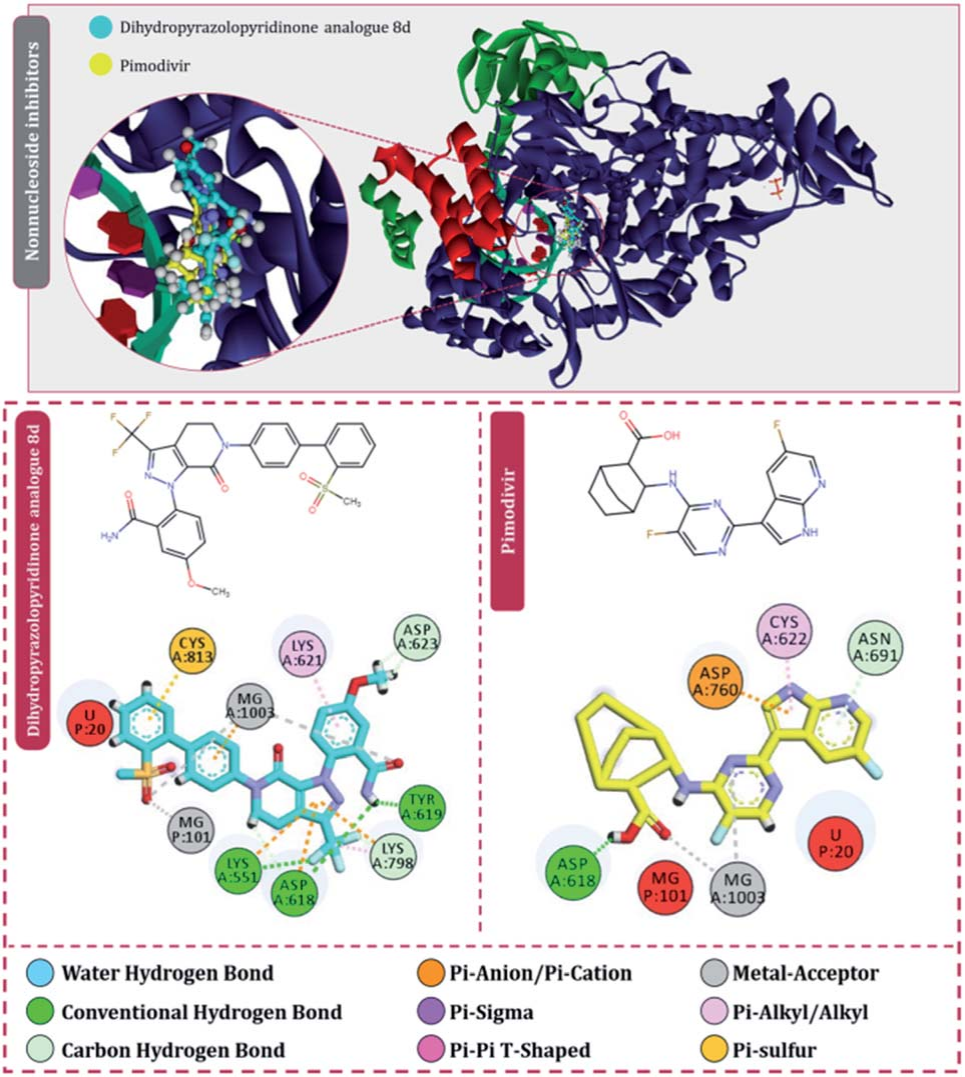
Interaction of nonnucleoside/nonnucleotide analogues, pimodivir and dihydropyrazolopyridinone analogue 8d with RdRp of SARS-CoV2.

Moving to the next group of RdRp inhibitors, *viz* nonnucleotide/nonnucleoside inhibitors, pimodivir was the top-scoring compound with the docking score of −7.18 kcal mol^−1^ and dihydropyrazolopyridinone analogue 8d was the second-best compound with the docking score of −6.98 kcal mol^−1^. The amino acid interaction profiles of these top hits with RdRp of SARS-CoV2 are shown in Fig. 5. The profile of dihydropyrazolopyridinone analogue 8d is much more interactive than the profile exhibited by pimodivir. Dihydropyrazolopyridinone analogue 8d interacts with Lys551, Asp618, and Tyr619 by making hydrogen bonds, Cys813 forms pi–anion interaction, Lys621 forms pi–pi interaction while Asp623 and Lys798 form a carbon–hydrogen bond, in total this drug forms interactions with seven different amino acids. On the other hand, pimodivir interacts with only four amino acids as follows, Asp618 by making hydrogen bond, Cys622 making pi–alkyl interaction, Asn691 making carbon–hydrogen bond formation, and Asp760 making pi–anion interaction. Pimodivir is originally known to inhibit RdRp of influenza A virus^47^ and efforts are being made to make its work for SARS-CoV2. However, there is a scarcity of clinical trial reports regarding its effectiveness on SARS-CoV2. Its poor interaction profile with the amino acids of SARS-CoV2 RdRp, despite its impressive docking score, might have ruled out the possibility for its effective use in the control of SARS-CoV2. In the later part of the manuscript, the MD simulation profile of this drug will shed more light on the same proposition. Dihydropyrazolopyridinone analogue 8d is known to be the inhibitor of A1 adenosine receptor, cyclin-dependent protein kinase-2 (cdk-2) and human nicotinamide phosphoribosyltransferase (NAMPT), along with, there are widely reported properties of this drug to be antimicrobial, anti-inflammatory, anticancer and antiplatelet.^48^ However, to the best of our knowledge, there are no reports of this compound being used as an inhibitor of RdRp. Though its efficacy to interact with RdRp of SARS-CoV2 is studied in detail under simulated conditions and is represented in the latter part of this paper. Next RdRp inhibitors of this group with reasonable docking scores are grazoprevir (−6.34 kcal mol^−1^) and radalbuvir (−6.28 kcal mol^−1^), which are twelfth and thirteenth overall (Table 1). The effectiveness of grazoprevir is theoretically predicted against RdRp of SARS-CoV2 by Behera and group,^49^ and there are other reports too predicting the effective interaction of this drug with RdRp of SARS-CoV2,^50^ however, to the best of our knowledge, there are no *in vitro* or trails of patients conducted for treating SARS-CoV2 with this drug. Grazoprevir is originally known to inhibit the infection of chronic hepatitis C infection.^51^ Radalbuvir is a reported inhibitor of hepatitis C NS5B polymerase and is currently in phase II clinical trials for oral use.^52^

Molecular docking assessment allowed screening of the top two nucleoside/nucleotide inhibitors identified as ribavirin 5′-triphosphate and favipiravir-RTP, while the top two nonnucleoside/nonnucleotide inhibitors screened were pimodivir and dihydropyrazolopyridinone analogue 8d. These four screened drugs were then analysed for MM-GBSA post docking assessment, which is the end-point binding energy change calculation. This assessment provides more dependable and reliable values of ionic, hydrophilic and hydrophobic attractions of the protein–ligand intricate. The Δ*G*bind energy conveyed from MM-GBSA assessment is the residual value when entropy value is subtracted from enthalpy, the value in the negative range shows the interaction between ligand and protein is spontaneous. In short, a more negative value indicates stronger binding and therefore Δ*G*Bind of MM-GBSA is used to estimate relative binding affinity for a list of ligands (reported in kcal mol^−1^). The binding energy change profiles in forms of MM-GBSA values of all the four screened drugs during their interaction with RdRp of SARS-CoV2 is tended to in Table 2. Δ*G*bind is most crucial to be viewed, where ribavirin 5′-triphosphate tops the list with a value of −12.34 kcal mol^−1^, followed by favipiravir-RTP (−10.43 kcal mol^−1^), followed by dihydropyrazolopyridinone analogue 8d (−6.93 kcal mol^−1^) and with pimodivir standing lowest with the value equal to −5.62 kcal mol^−1^. In addition to the total energy, the contributions of the total energy from different components such as hydrogen-bonding correction, Coulomb energy, pi–pi stacking correction, van der Waals energy and lipophilic energy are also provided in Table 2. All these parameters conclusively help to determine the secondary ranking of these four compounds in the order from the highest to the lowest as ribavirin 5′-triphosphate, favipiravir-RTP, dihydropyrazolopyridinone analogue 8d and pimodivir. The sub-atomic mechanics energies joined with the Poisson–Boltzmann or summed up Born and surface territory continuum solvation commonly referred to as MM-PBSA and MM-GBSA strategies are mainstream ways to deal with the spontaneity of ligand–receptor interactions. They are normally founded on sub-atomic elements simulations of the receptor–ligand complex and in this way possess both precision and computational exertion between exact scoring and severe catalytic bother strategies.^53^ The prime module of Maestro used in this study performs its own simulation based on the ‘best-docked protein–ligand pose’ by using the highly robust VSGB 2.0 energy model.^54^ MM-GBSA is applied to an enormous number of protein–ligand interaction frameworks with tremendous success to validate the outcomes of molecular docking.^55–59^

**Table 2:** MM-GBSA profile of top hits^a^

Ligand	Δ*G*Bind (kcal mol^−1^)	Δ*G*Coulomb (kcal mol^−1^)	Δ*G*Hbond (kcal mol^−1^)	Δ*G*Lipo (kcal mol^−1^)	Δ*G*vdW (kcal mol^−1^)
Ribavirin 5′-triphosphate	−12.34	−52.26	−3.56	−10.13	−42.45
Favipiravir-RTP	−10.43	−62.15	−3.65	−4.56	−41.59
Pimodivir	−5.62	−46.49	−0.89	−11.45	−38.25
Dihydropyrazolopyridinone analogue 8d	−6.93	−55.35	−0.77	−10.26	−40.23

a Δ*G*Bind – binding energy, Δ*G*Coulomb – Coulomb energy, Δ*G*Hbond – hydrogen-bonding correction, Δ*G*Lipo – lipophilic energy and Δ*G*vdW – van der Waals energy.

### MD simulation assessment

The surety proposed by the docking assessment for interaction occurring between ligand and protein would actually translate into reality or not is evaluated robustly by MD simulations. The MD simulations will reassure the interaction length, interaction types occurring as predicted by docking or not. Docking will only predict the type of interaction that may occur between a protein with a ligand for a given pose, but the strength of the interaction is not predicted, MD simulations help to overcome this limitation of molecular docking. Moreover, the stability of the best-docked pose of the ligand with respect to its interacting protein can also be evaluated over a course of time under MD simulations.^10,30,60–62^

**Fig. 6:**
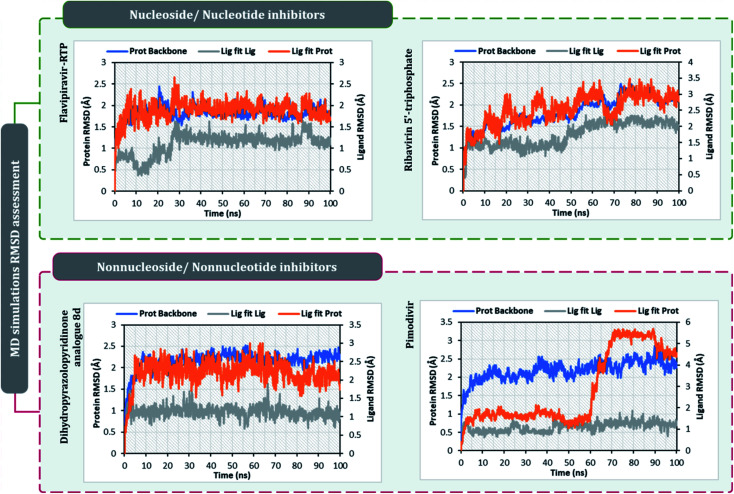
RMSD profiles of SARS-CoV2's RdRp complexes individually with ribavirin 5′-triphosphate, favipiravir-RTP, pimodivir and dihydropyrazolopyridinone analogue 8d obtained from the 100 ns MD simulation.

After performing post-docking analysis of all the RdRp inhibitors, a total of four drugs, of which two being nucleoside/nucleotide analog inhibitor and other two being nonnucleotide/nonnucleoside inhibitors, where RdRp of SARS-CoV2 in individual complexes each with ribavirin 5′-triphosphate, favipiravir-RTP, dihydropyrazolopyridinone analogue 8d and pimodivir were analyzed by performing 100 ns MD simulation runs. The first assessment performed was to access the Root Mean Square Deviations (RMSD) for each drug-RdRp complex. Here, there are two main analysis (i) protein RMSD (ii) ligand RMSD with respect to protein. Fig. 6 is the graph portraying RMSD deviations of all the complexes under study. The left *Y*-axis addresses the sections of the protein during MD reproductions, a piece of which is additionally protein equilibration. During surveying the trajectories of MD simulation, the RMSD assessment must be performed for assessing the movement and structural change in native 3D protein structure and orientation with the reference to the native structural frame. The protein backbone RMSD changes in the range of 1–3 Å are completely normal for little, globular proteins, however, the range may increase for larger proteins. Changes, a lot bigger than that, notwithstanding, show that the protein is going through a huge conformational change during the MD reproductions. It is additionally significant that the protein RMSD values stabilize after few tens of ns around a fixed value which also suggests the protein as equilibrated properly. Under the current study, it is observed for the protein RdRp complexes with all the screened drugs, individually reaching the equilibration before 10 ns attaining a constant RMSD value which, never exceeds 3 Å despite relatively being a large protein (Fig. 6). The right *Y*-axis denotes the RMSD of the ligand, which is a measure of how stable the ligand is in the docked pose at the catalytic site of RdRp. ‘Lig fit Prot’ shows the RMSD of a ligand when the protein–ligand complex is first adjusted on the protein backbone of the reference and afterward the RMSD of the ligand is estimated. It is believed that the value of ‘Lig fit Prot’ reaching marginally greater than the protein's RMSD are viewed as agreeable, but when this value is fundamentally bigger than it signifies the orientation of ligand predicted by docking is unstable and therefore ligand reorients to acquire a stable conformation. Under the current study, the ‘Lig fit Prot’ values of ribavirin 5′-triphosphate, favipiravir-RTP and dihydropyrazolopyridinone analogue 8d is acceptable but not acceptable for pimodivir as the values peaks to 5.8 Å which is almost double than that of protein RMSD (Fig. 6). Thus, from this, it can be concluded that pimodivir indecisively interacts with RdRp of SARS-CoV2 despite showing an acceptable docking score.

**Fig. 7:**
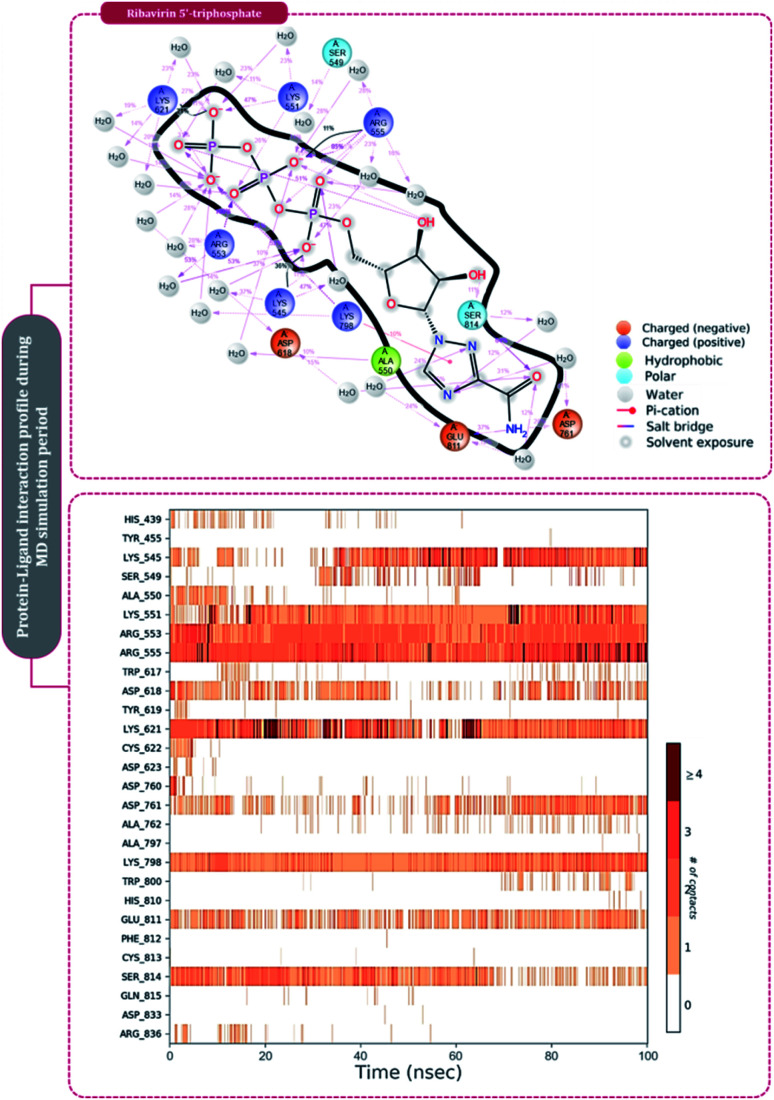
Amino acid interaction timeline profile along the course of 100 ns MD simulation developed by the interaction of ribavirin 5′-triphosphate with RdRp of SARS-CoV2.

**Fig. 8:**
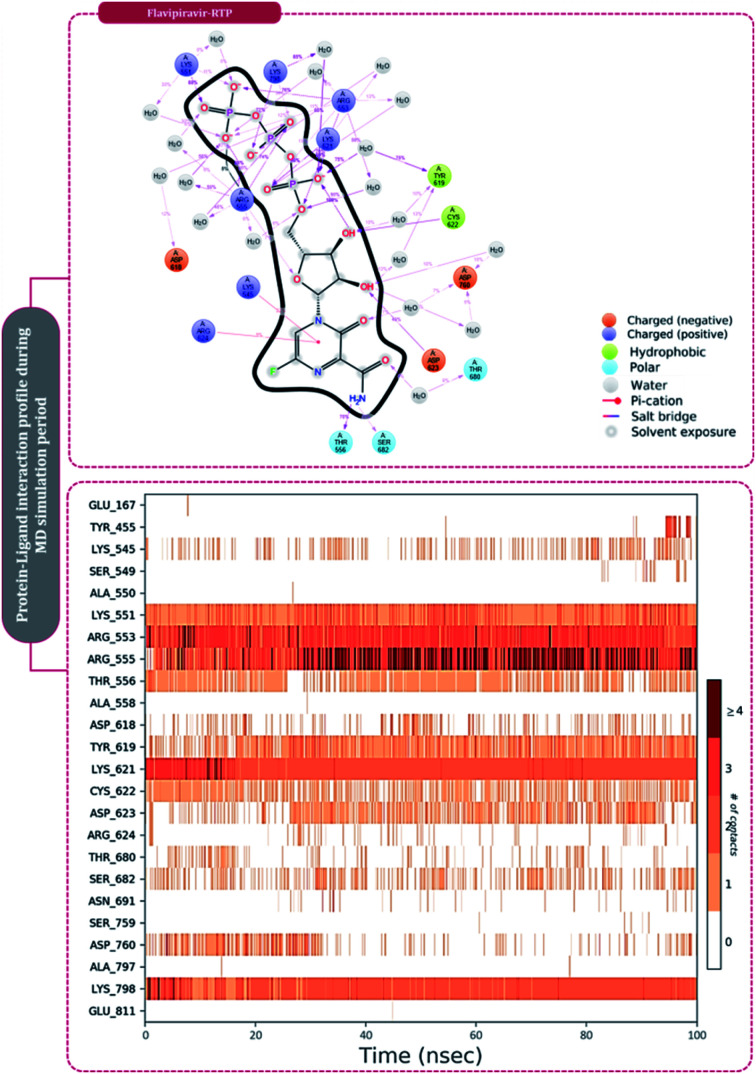
Amino acid interaction timeline profile along the course of 100 ns MD simulation developed by the interaction of favipiravir-RTP with RdRp of SARS-CoV2.

**Fig. 9:**
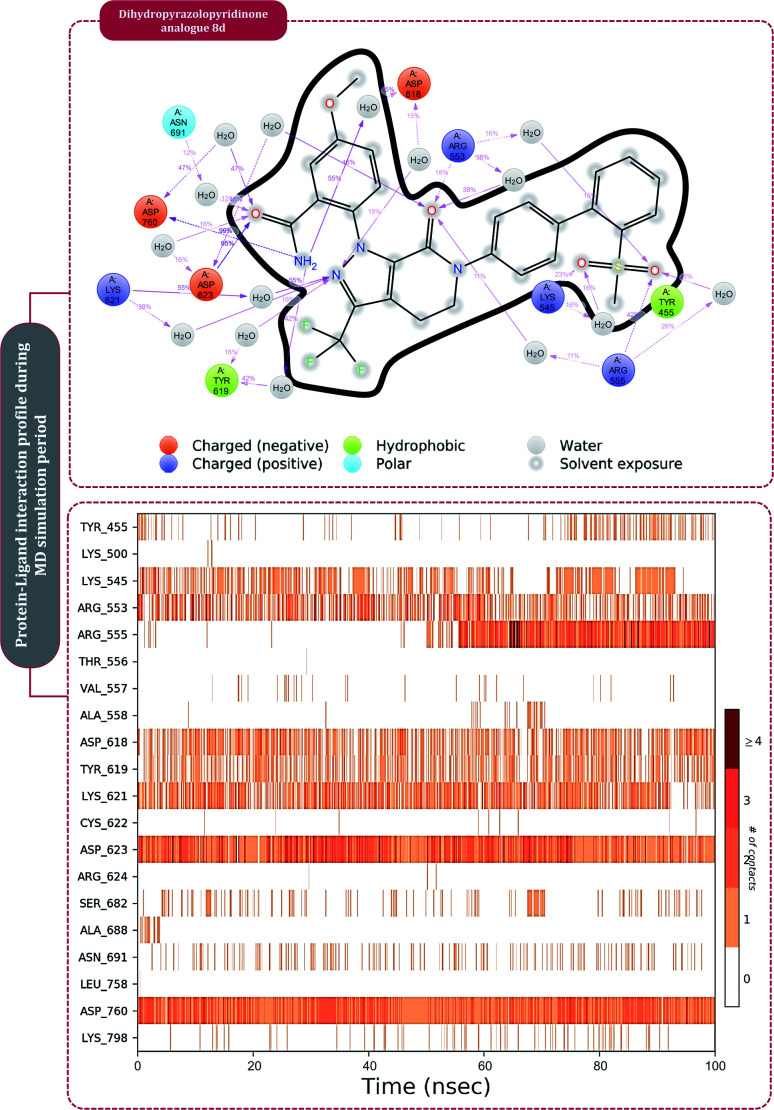
Amino acid interaction timeline profile along the course of 100 ns MD simulation developed by the interaction of dihydropyrazolopyridinone analogue 8d with RdRp of SARS-CoV2.

**Fig. 10:**
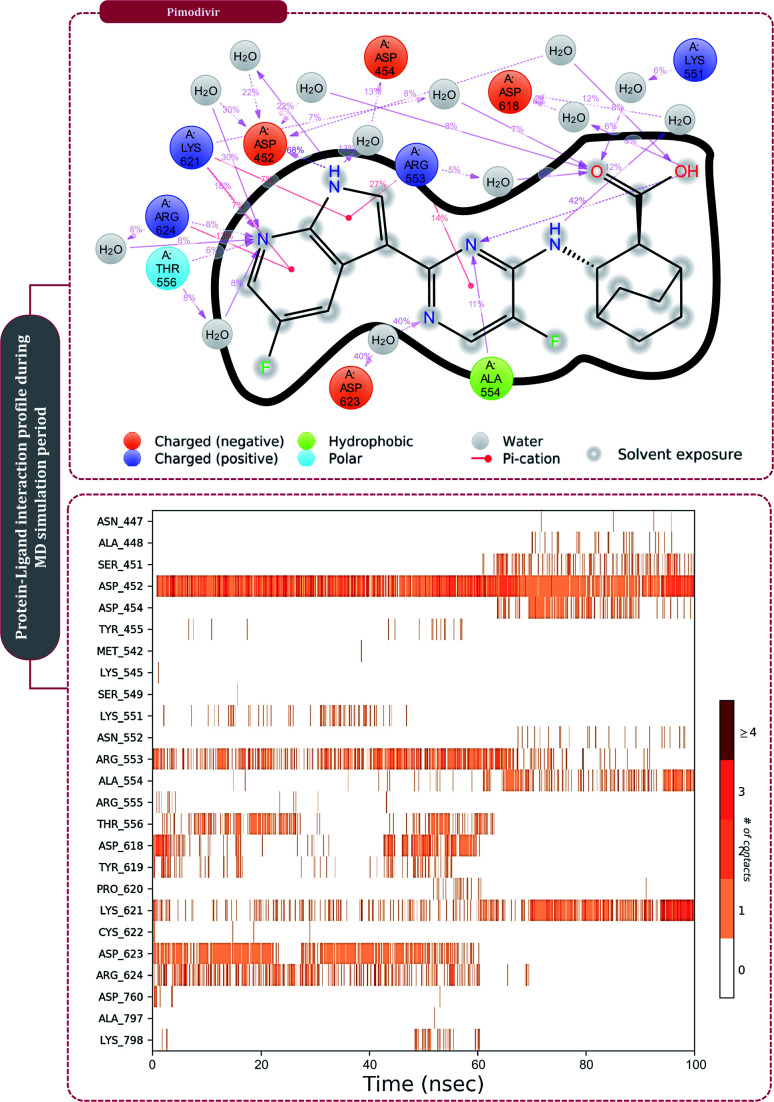
Amino acid interaction timeline profile along the course of 100 ns MD simulation developed by the interaction of pimodivir with RdRp of SARS-CoV2.

**Fig. 11:**
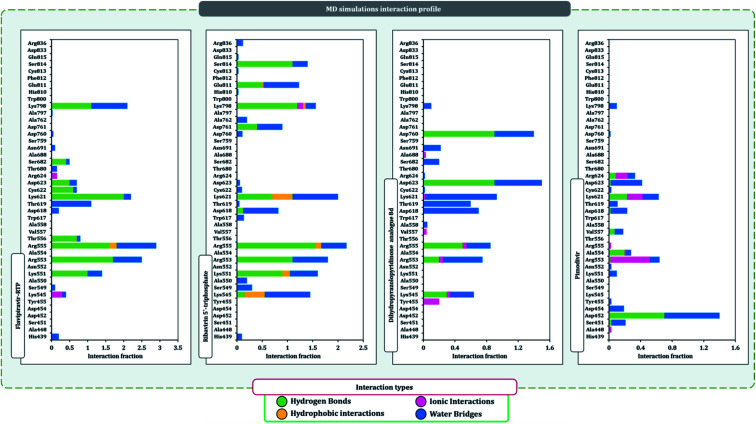
SARS-CoV2-RdRp amino acid interaction types exhibited by top inhibitors during 100 ns MD simulation.

The protein–ligand interaction timeline of ribavirin 5′-triphosphate with the amino acids of SARS-CoV2 RdRp is represented in Fig. 7. The important amino acids with which it makes regular contacts during the 100 ns simulation run are Lys545, Ala550, Lys551, Arg553, Arg555, Asp618, Lys621, Asp761, Lys798, Glu811 and Ser814. The only amino acid that was predicted to strongly interact as per docking assessment but weekly interacted during simulation is Trp800. On the contrary strong interactions formed during MD simulations but were not predicted by docking assessments are with Lys545, Lys551, Arg553, Arg555, Asp618, Asp761, and Lys798 (Fig. 4). RMSD assessment along with protein–ligand contact profile suggests that ribavirin 5′-triphosphate poses tremendous capability to interact with amino acids in the catalytic site of RdRp and inhibit its function. Fig. 8 shows the protein–ligand interaction timeline of favipiravir-RTP, where it is observed that Lys545, Lys551, Arg553 Arg555, Asp618, Lys621, Asp761, Lys798, Glu811 and Ser814. Again, for this case the only amino acid that was predicted to strongly interact as per docking assessment but weekly interacted during simulation is Trp800. On the contrary strong interactions formed during MD simulations but were not predicted by the docking, assessments are with Lys545, Lys551, and Lys798 (Fig. 4). Like ribavirin 5′-triphosphate, even for favipiravir-RTP with the viewpoint based on RMSD assessment and protein–ligand interaction timeline, favipiravir-RTP here is proposed to strongly interact with the amino acids in the catalytic site of RdRp and inhibit its function. Moreover, as per the significant recent publication by Jiang and colleagues in 2021,^63^ the essential amino acids that play a significant role for binding of RdRp inhibitors with RdRp of SARS-CoV2 are, Lys545, Arg555, Asp623, Ser 682 and Asn691. All these amino acids significantly interact with both ribavirin 5′-triphosphate and favipiravir-RTP, throughout the MD simulation (Fig. 7 and 8). Protein–ligand interaction timeline for dihydropyrazolopyridinone analogue 8d interacting with SARS-CoV2's RdRp is represented in Fig. 9, and from the profiles so generated, this drug during simulation is seemed to strongly interact with Lys545, Arg553, Asp618, Tyr619, Lys621, Asp623, Ser682, Asn691 and Asp760. Moreover, dihydropyrazolopyridinone analogue 8d, could interact with all the important amino acids (Fig. 9) that are needed for the inhibitor for RdRp's inhibition as per Jiang and colleagues in 2021.^63^ Dihydropyrazolopyridinone analogue 8d during MD simulations showed much promising interactions throughout the time of simulation than the interaction predicted by molecular docking (Fig. 5). From the assessment dihydropyrazolopyridinone analogue 8d can be a strong drug to inhibit RdRp for which the clinical trials are not yet reported. Fig. 10 shows the protein–ligand interaction timeline for pimodivir interacting with SARS-CoV2's RdRp. The interaction profile is relatively poor when compared to the previous three drugs studied for the same assessment. Pimodivir is forming interaction with only one amino acid on constant reliable grounds that is with Asp452. Moreover, the interaction profile for pimodivir as predicted by molecular docking is equally poor (Fig. 5). From poor MM-GBASA Δ*G*bind score to poor RMSD profile during MD simulations and lastly with poor interaction with amino acids during MD simulations, pimodivir cannot be classified as the inhibitor of RdRp, based on this theoretical study. The type of interaction involved for the interacting amino acid of RdRp with each drug under study during the MD simulations is represented in Fig. 11. This interaction types are classified into four kinds: hydrogen bonds, hydrophobic connections, ionic contacts, and water bridges, which can be researched through the graphical representation of ‘Simulation Interactions Diagram’. The stacked bar traces are normalized cumulative interaction profile: for example, an assessment of 0.8 suggests that 80% of the time during the simulation, the corresponding interaction remains durable. Characteristics over 1.0 are possible as some amino acids may have more than one type of contact of the equivalent subtype with the ligand. Thus, on the whole, it can be predicted that from all 60 drugs under clinical trials, ribavirin 5′-triphosphate, favipiravir-RTP and dihydropyrazolopyridinone analogue 8d can serve as the potent inhibitor for RdRp. Lack of clinical trials with dihydropyrazolopyridinone analogue 8d, makes us leave with only two potent drugs, respectively, as ribavirin 5′-triphosphate and favipiravir-RTP.

It is practically difficult to work with SARS-CoV2 in the laboratory as it requires ethical permissions and special safety precautions. Under such a scenario rigorous *in silico* workflow involving docking, MD simulation, and MM-GBSA assessments is helping us predict the behavior of drugs for SARS-CoV2 with high accuracy. Under such a scenario, large volumes of data developed using computational study are brought to researchers' domain having facility to work in the sophisticated lab with SARS-CoV2, that can help validate computational predictions with laboratory experiments saving a tremendous heap of time (Fig. 11).

## Conclusions

As RdRp is the enzyme commonly found in all RNA viruses but is absent in humans, and medications in the form of RdRp inhibitors for the other RNA viruses are already known and are currently repurposed specifically for RdRp of SARS-CoV2. Using rigorous computational workflow, a total of over 60 different RdRp inhibitors already under clinical trials for combating other RNA viruses are targeted specifically against the RdRp of SARS-CoV2, making this strategy to be called ‘drug repurposing. These inhibitors fall into two main groups (i) nucleoside/nucleotide analogues and (ii) nonnucleoside/nonnucleotide analogues. The results of docking showed ribavirin 5′-triphosphate and favipiravir ribonucleoside triphosphate (favipiravir-RTP) to be the top two nucleotide analogues while pimodivir and dihydropyrazolopyridinone analogue 8d to be the top two nonnucleoside/nonnucleotide analogues. Pimodivir later showed a poor MM-GBSA Δ*G*bind score and MD simulations showed that it poorly interacted with amino acids of SARS-CoV2's RdRp, and so the possibility to be a potent RdRp is ruled out. While the lack of clinical trials with dihydropyrazolopyridinone analogue 8d, makes us live with only two potent drugs, ribavirin 5′-triphosphate and favipiravir-RTP, that can be augmented for further clinical trials to develop a reliable mode to treat SARS-CoV2.

## Author contributions

D. G. conceived designed the experiments, performed the experiments, analysed the data, and wrote the manuscript.

## Conflicts of interest

There are no conflicts to declare.

## Supplementary Material

RA-011-D1RA04460E-s001
