# Supplementary material for: Comparative assessment of RNA-dependent RNA polymerase (RdRp) inhibitors under clinical trials to control SARS-CoV2 using rigorous computational workflow
Source: RSC Adv. 2021 Sep 2;11(46):29015–28. doi: 10.1039/d1ra04460e (PMC9038185; doi:10.1039/d1ra04460e)
Supplement: RA-011-D1RA04460E-s001 [file RA-011-D1RA04460E-s001.pdf]

Electronic Supplementary Material (ESI) for RSC Advances.
This journal is © The Royal Society of Chemistry 2021

Supplementary material

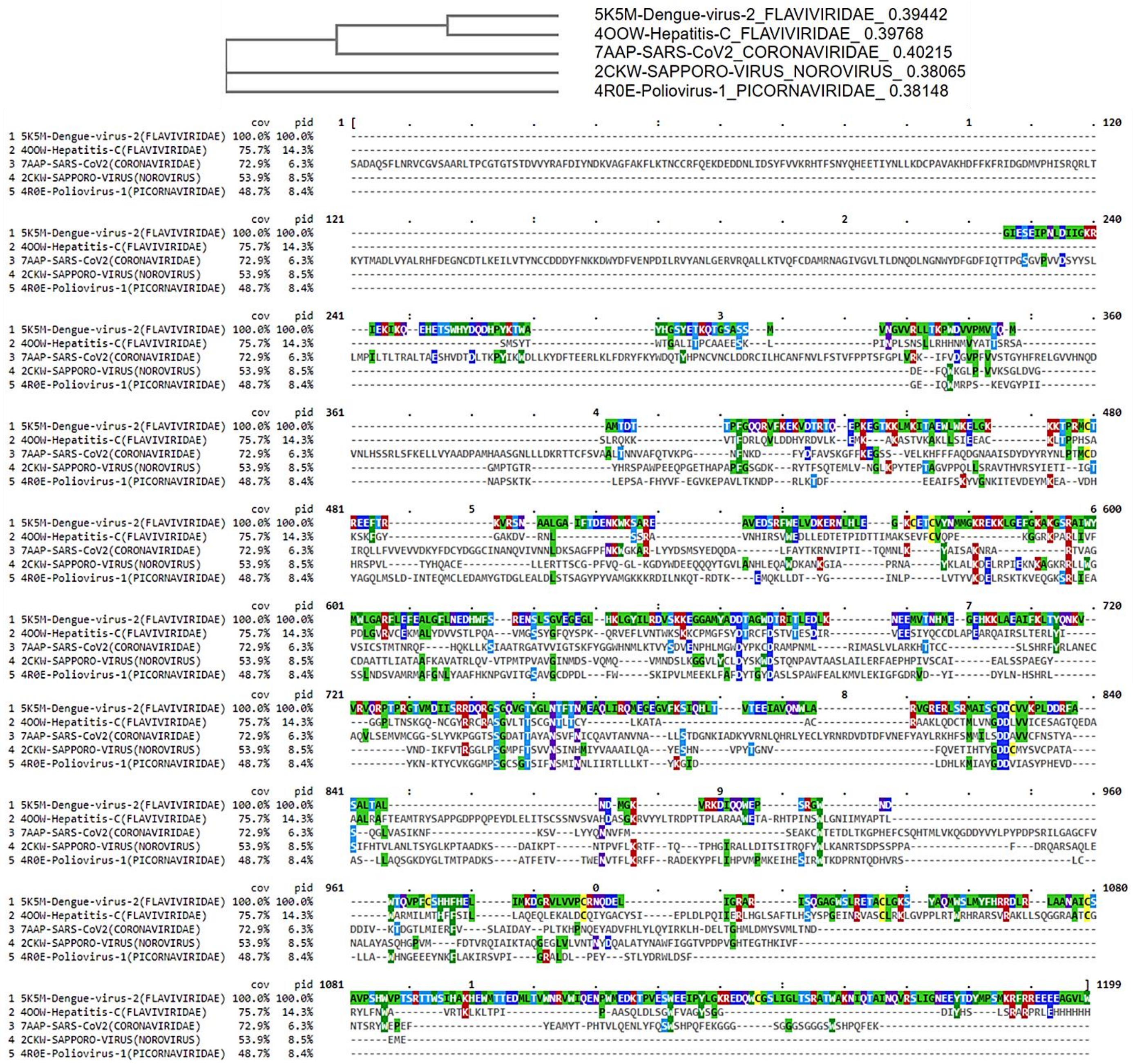

Fig. S1 Phylogenetic analysis and Multiple sequence of RdRps from various +ssRNA viral family representative viruses.
